# A Comparison between Two Intervals of Daily Total End Range Time for Treatment of Proximal Interphalangeal Joint Flexion Contracture Using an Elastic Tension Digital Neoprene Orthosis

**DOI:** 10.3390/jcm12051987

**Published:** 2023-03-02

**Authors:** Vicenç Punsola-Izard, Aroa Casado, Nuria Carnicero, Elena Ozaes-Lara, Judit Mendieta-Zamora, Gemma Romera-Orfila, Karen S. Schultz, Manuel Llusà

**Affiliations:** 1 Hand Therapy Barcelona Physical Therapy and Clinical Investigation Center, 08010 Barcelona, Spain; 2 Physiotherapy Department, Gimbernat School of Physical Therapy, 08174 Barcelona, Spain; 3 Department of Evolutionary Biology, Ecology and Environmental Sciences, University of Barcelona, 08007 Barcelona, Spain; 4 Karen Schultz Hand and Upper Limb Strategies (KSHULS), Littleton, CO 80120, USA; 5 Unit of Human Anatomy and Embryology, University of Barcelona, 08036 Barcelona, Spain

**Keywords:** proximal interphalangeal joint, finger treatment, orthosis, hand therapy, flexion contracture, total end range time

## Abstract

Focusing on fingers with proximal interphalangeal joint flexion contractures, this study seeks to determine whether significant differences exist between the joint passive range of motion PROM improvement when receiving higher doses of daily total end range time (TERT) compared to those that receive lower doses. The study randomized a parallel group of fifty-seven fingers in fifty patients with concealed allocation and assessor blinding. Divided into two groups receiving different doses of daily total end range time with an elastic tension digital neoprene orthosis, they also participated in an identical exercise program. Patients reported orthosis wear time, and the researchers performed goniometric measurements at every session during the three-week period. The primary outcome related the time patients wore the orthosis to the degrees of improvement in PROM extension. Compared to group B (daily TERT of twelve hours), group A (TERT, twenty+ hours) showed a statistically significant greater improvement in PROM after three weeks of treatment. Group A improved by a mean of 29° compared to group B’s mean of 19° improvement. This study provides evidence that a higher dose of daily TERT can generate better results in the treatment of the proximal interphalangeal joint flexion contractures.

## 1. Introduction

Proximal interphalangeal joint (PIPJ) flexion contracture is a frequent complication after in-hand conditions that have a neurologic, inflammatory, trauma, or post-surgical diagnosis [1]. Conservative treatment of this pathology often consists of positioning the contracted joint at end range for prolonged periods of time [2,3]. The literature supports the use of orthoses to position the joint at the end range, and, in this manner, to deliver tissue tension as a means to alter the length of restricting structures [2,4,5].

The optimal dose of tissue tension that end range position imparts has two main variables: the amount of time the joint remains at end range and the amount of force directed to the affected joint tissues [2,6,7]. Light introduced this concept of a force/time combination in conjunction with his studies of low load prolonged stress (LLPS) and contrasted it with the widely practiced option of applying high load brief stress (HLBS) [3]. Light’s studies demonstrated that LLPS provided superior outcomes when compared to HLBS in restoring passive range of motion (PROM). While an increase in the amount of time may have a positive effect, an increase in the amount of force can easily go beyond the limits imposed by pain and can cause inflammation and tissue damage [8]. 

Flowers and LaStayo developed further evidence of the positive impact of time when maintaining a joint at available end range. They chose casting as a treatment approach because it creates a sustained twenty-four hour a day joint position for all patients [2]. This study helped to develop the term total end range time (TERT) and described that “the amount of improvement in PROM of a stiff joint is directly proportional to the amount of time the joint is positioned at its end range, or TERT”. Researchers and clinicians, continuing to look at the effect of end range positioning, pointed out that TERT can describe the amount of time a joint sits at end range each day (i.e., ten hours a day) or the cumulative orthosis wear time (i.e., sixteen hours a day for three months) [4,5]. No one has conducted research into the therapeutic indications for different force intensities [7]. 

Many articles [8,9,10] describe the use of serial casting orthoses (SCOs) as an effective tool to improve PIPJ flexion contractures. Most hand therapists have clinical evidence of this concept. However, no studies describe either a specific dose application regimen or demonstrate the validity of the approach with well structured research using a clinical series of subjects. Rather, the literature is replete with anecdotal evidence and expert opinion [7]. 

SCOs have some potential advantages and drawbacks. SCOs optimize time because the patient wears them twenty-four hours a day. However, SCOs cannot create the most effective ongoing end range position. As Flowers described, immediately upon orthosis removal, the joint gained a few more degrees of PROM [2]. SCOs cannot capitalize on increases in PROM until the therapist fabricates a new cast [11]. In addition, SCOs require regular therapy attendance that can prove difficult to achieve in many populations [12]. Finally, some concern exists that such continuous immobilization might either foster adhesions or cause soft tissue degradation [5,13,14,15]. Importantly, Prosser [4] arrived at the conclusion that treatment of a stiff PIPJ with orthosis application requires four-point-three months duration to achieve a ROM plateau. Clinical experience shows that many patients will find serial casting impractical for this time span. No one has studied the application of serial casting over a four-month period. Given these serial casting issues, we need to explore other therapeutic tools to achieve the needed increase in time.

Elastic tension orthoses (ETOs) offer yet other benefits and drawbacks. This method has the advantage of promoting the constant progression of end range and maximum tissue tension [5,16]. As tissue lengthens, the orthosis takes advantage of this increased length and progresses the tissue to ever greater lengths. ETOs also have the characteristic of allowing intermittent removal. This avoids any potential secondary effects of prolonged immobilization. This approach allows patients to increase the cumulative TERT because they can use it for a longer duration. However, removal also leads to a significant reduction in the “dose” of daily TERT.

Prosser and Glasgow [4,5,17] explored stress dose application to tissue via the amount of time of orthotic use. Prosser explored application of TERT with two different types of ETOs [4]. She did not find a difference between them, but she found a statistically significant correlation between the extension improvement and TERT. Glasgow explored the effect of daily TERT, and she found evidence of its positive effect [5]. In the Glasgow study of two amounts of end range time, group B (six to twelve hours of daily TERT) improved by nearly double the degrees of group A (zero to six hours of daily TERT). In another study of PIPJ flexion contracture treatment [17], she again explored the effect of the TERT dose using spring wire ETOs. She instructed Group A to wear the orthosis for six to twelve hours and group B to use the orthosis from twelve to sixteen hours. In this study, no subject in group B was able to wear the orthosis more than twelve hours. Based on opinion, the author concluded that most of the patients found that the use of the spring wire orthotic limited their activities of daily living (ADL), and thus they could not achieve a wear time exceeding twelve hours. However, she did not offer evidence in connection to the actual reason for inability to wear the orthosis for more than twelve hours. 

For many years, therapists have used spring wire orthoses to treat PIPJ flexion contractures [1,18,19,20]. These orthosis designs have a mobile axis of rotation and a fixed lever arm length. This configuration results in a shift of the orthosis force on the finger as the joint angle changes [16]. The combination of the lever arm issue and the relatively small skin–orthosis interface appears to contribute to the difficulty with wear over long periods of time due to discomfort [17]. Since 2012, no published studies have achieved a daily TERT greater than twelve hours. The use of soft materials using a circumferential design may offer a good option to facilitate a TERT increase [21,22]. Soft materials, such as neoprene, with a large skin contact area, provide high levels of pressure distribution and a corresponding increase in comfort [22,23,24,25]. Punsola-Izard [23] proposed a soft custom orthosis method using neoprene with a design specific to an individual patient, the elastic tension digital neoprene orthosis (ETDNO). Punsola-Izard [21] demonstrated how, in a three-week period, an ETDNO improved PIPJ extension and obtained a daily TERT of nearly twenty-two hours. These findings established a means to study long periods of joint extension at end range. He also described a clinical case of a patient with a 45° PIP flexion contracture who used an ETDNO serial elastic tension protocol to achieve full extension [22]. This current study seeks to apply this ETDNO orthosis method to explore the effect of time on the treatment of PIP flexion contractures. 

### Hypotheses

A twenty to more than twenty-two-hour period of PIPJ extension at end range position will be more effective in increasing extension PROM than a period of ten to fourteen hours.

## 2. Materials and Methods

### 2.1. Study Design

**Figure 1:**
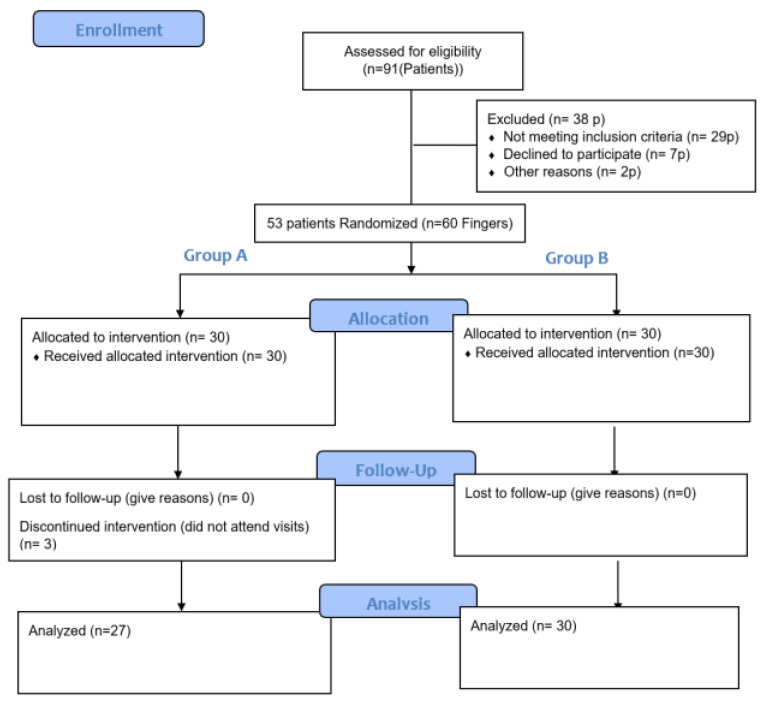
CONSORT 2010 Flow Diagram.

We conducted a randomized parallel-group clinical trial (Figure 1).

### 2.2. Setting

Assessment and treatment procedures took place in Hand Therapy Barcelona Physical Therapy and Clinical Investigation Center and at the Trauma Unit, Dr. Casañas Clinic at Teknon Clinic of Barcelona.

### 2.3. Study Participants

From July 2022 to December 2022, ninety-one patients presented with PIPJ motion impairment. The patients who met the inclusion criteria presented with flexion contractures of the PIPJ from trauma or post-surgical complications (Table 1). We also included patients with long standing flexion contractures or contractures greater than 45°. We excluded patients with acute tendon injuries or fractures, inflammatory conditions, PIPJ replacements, or Dupuytren conservative treatments. We excluded patients lacking active PIPJ extension because, as Prosser stated, “passive extension cannot be maintained if there is inadequate active extension”. From these ninety-one patients, we initially selected fifty-three patients, representing sixty PIPJs that fit the inclusion criteria. Three patients dropped out of the study because they did not attend their appointments. This left fifty patients with fifty-seven involved PIP joints. Thirty-eight patients were excluded because they failed to meet the inclusion criteria.

### 2.4. Intervention Design

The patients participated in a newly designed program to treat PIP flexion contracture. The first part of this program involved application of a passive extension device to improve extension of the PIPJ. The second part of the program incorporated an exercise program to maintain or improve flexion of the PIPJ. We selected the ETDNO extension orthosis because it is user friendly. The soft neoprene material makes it comfortable and low-profile for the finger during function. The choice of the ETDNO extension orthosis facilitated easy orthosis removal so the patient could perform flexion exercises without difficulty.

The primary researcher evaluated all patients presenting to the clinic who demonstrated PIPJ stiffness. A researcher collaborator without direct participation in the clinical aspects of the study produced a computer-generated set of random numbers to create an allocation sequence. The researcher used this to randomize each subject into one of two groups. Patients in Group A used the ETDNO for twenty to more than twenty-two hours a day, while patients in Group B used the ETDNO for ten to fourteen hours a day. Group A consisted of twenty-four patients with twenty-seven PIPJs. Group B consisted of twenty-six patients with thirty PIPJs (Table 1).

**Figure 2:**
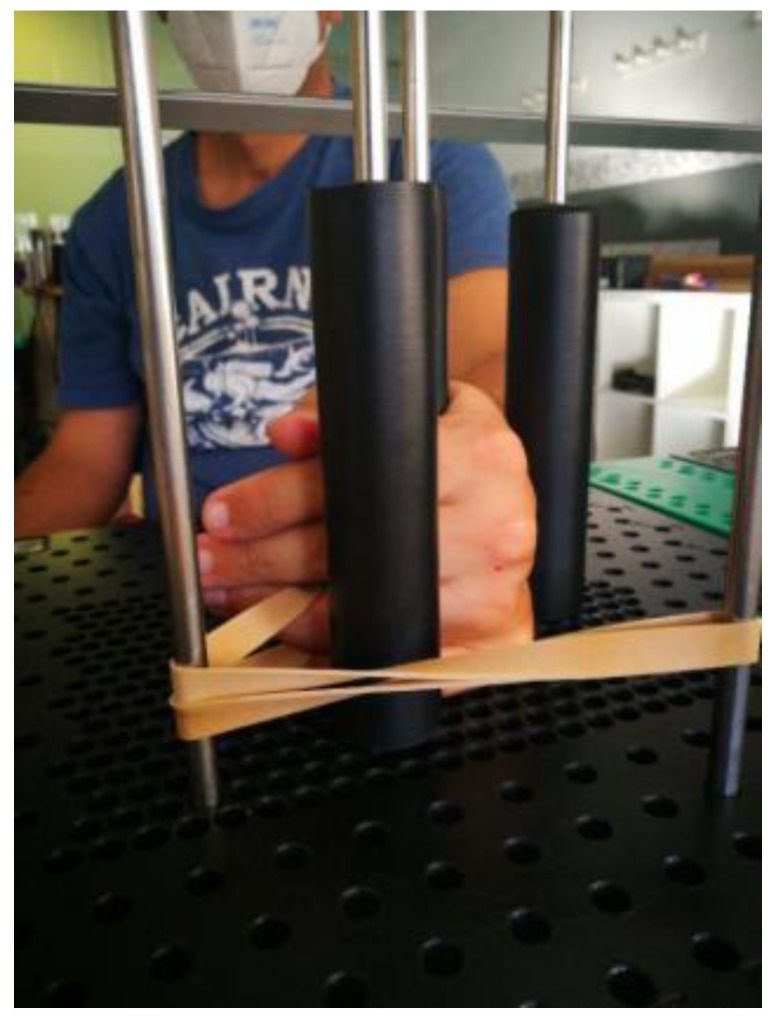
500 g extension force at 90° applied for fifteen minutes at the head of P2 using a *MAPS* Therapy Pegboard.

During the initial visit to the clinic, the primary researcher gathered demographic data, including age and gender and medical history, including type of injury, time since the contracture, and previous treatments. To control for tissue temperature, the patients underwent hot pack application for fifteen minutes in a resting end range extension position prior to taking initial measurements. The researcher then performed a modified weeks test (MWT), as described by Flowers [6,17] to attempt to establish a prognosis for the effectiveness of treatment. After the hot pack application, the researcher measured PROM in both flexion and extension. He then preconditioned the PIPJ using a MAPS therapy device (Figure 2) with an extension force of five-hundred grams applied at the head of the second phalanx at 90° for fifteen minutes. Following this procedure, the researcher again measured passive extension to complete the MWT.

The researcher measured each patient’s finger circumferences and length as required for the construction of the ETDNO. He then fabricated a custom ETDNO for all subjects. Each ETDNO matched the finger flexion contracture position.

Our concern about losing flexion while patients engaged in extension focused treatment motivated us to instruct the patients in incorporate short recurrent intervals of flexion exercise. We instructed all patients to remove the ETDNO at least four times a day to exercise the finger using an active assisted flexion approach for twenty minutes. An additional caveat for the patients in Group A stated that the periods of removal could not exceed the ability to wear the orthosis for twenty/twenty-two or more than these hours a day. After exercise, patients in Group A could decide whether to put the ETDNO back on again or perform any chosen ADL activity without the orthosis for the rest of the daily ETDNO free hours. Patients in group B received encouragement to freely use their hands during the twelve hours without ETDNO use. Patients did not use any other treatment interventions. 

After the first visit, we scheduled the patients to attend the clinic once a week for three weeks. During each visit, the patients reported any difficulty related to the ETDNO use and also about their compliance with the TERT program. In addition, patients underwent measurement of finger PROM extension twice. We performed the initial measurement at the beginning of the session, just after the patient removed the ETDNO. Following the measurement, each patient exercised in flexion for twenty minutes. After exercise, we measured flexion PROM and repeated the extension PROM evaluation. We recorded the difference between the first and the second extension measurement and designated this difference as the “Contraction Test”. The goal of this measurement was to identify the tissue response after releasing extension force and focusing on flexion for twenty minutes.

As soon as the therapist measured an improvement greater than 10° extension at the ETDNO removal, the therapist made a new ETDNO at the new position. We understood that this new ETDNO imparted an increase in treatment dose by amplifying the force. Because this new ETDNO could cause discomfort until the finger adapted to the new tension, the patient received instruction as follows: “during the first forty-eight hours, if you experience any discomfort, remove the new ETDNO for one hour and use the previous ETDNO during that time. If discomfort occurs during the night, use the previous orthosis while sleeping”. 

### 2.5. Statistical Analysis

To establish a comparison between the two study groups, we carried out a normality study of the sample using the Shapiro–Wilk statistical test. The statistical analysis corroborated the distribution of the sample. Next, we applied, the non-parametric Mann-Whitney U statistical test to explore the intragroup and intergroup differences from the beginning to the end of the study at the third week.

For the calculation of the sample size, we considered a difference of fifteen degrees of mobility as the smallest effect for the optimal recovery of mobility. We anticipated a ten-degree standard deviation. Based on these values, a two-sided alpha error rate of 0.05, and a power of 80%, we calculated that the required sample size would be twelve subjects in the first group and nine in the second. We estimated a 30% dropout rate. Despite the sample size calculation, the authors of the study decided to increase the N by including all patients in the center who met the inclusion criteria.

## 3. Results

The evaluation of results focused on the amount of daily TERT and on the PROM outcomes. We also examined aspects of the qualitative measurements, including the MWT and the contraction test.

### 3.1. Daily Total End Range Time 

Via interview, we learned that patients in Group A obtained a mean of twenty and a half hours of daily TERT. Group B accomplished the daily TERT targets with a mean daily TERT of twelve hours. In Group A, seventeen fingers, or 63%, and, in group B, six fingers, or 20%, had no problem when using the ETDNO (Table 2).

### 3.2. Passive Range of Motion (PROM) 

The mean improvement after the first week was 18° for group A and 12.5° in group B. This represented 501° (52.84%) of the Potential Improvement to Neutral (PIN) in group A and 377° (41.24%) of PIN in group B. At the third week, the mean improvement in group A was 29° (83.22% of PIN) and 19° in group B (62.36% of PIN). To see absolute numbers, please refer to Table 3. Despite the prolonged doses of daily TERT in extension, the subjects gained flexion. Indeed, instead of exhibiting flexion loss, PIPJ flexion improved by a mean of 5.5° in group A and 4.2° in group B. Four patients in group A and five patients in group B lost flexion because they did not comply with the flexion exercises as instructed (Table 3 and Table 4).

The findings of this study support our hypotheses that a twenty to twenty-two or more hours period of joint extension at end range position will be more effective in increasing PIPJ extension PROM than a period of ten to fourteen hours with the joint extension at end range position. PIPJs receiving the higher level of TERT demonstrated improvement that is statistically significant when compared to those receiving the lower level of TERT.

### 3.3. Qualitative Tests

All patients in group A and group B initially presented with a positive MWT. There were no significant differences between group A and B. When we compared the range of Weeks test from the first day with the final result, we did not find a clinical or statistical correlation. Some of the joints had a very hard end feel at the beginning, but improved, while others had a softer end feel initially, but finally had a poor result. Taking this finding into consideration, the MWT does not appear to have a predictive value for our subject sample (Table 5). 

We initiated the contraction test at the end of the first week of treatment. All patients demonstrated a loss of extension PROM after undergoing the contraction test procedure. This extension loss occurred at the first week evaluation and also at the third week evaluation. We compared the contraction test values of group A and group B at the first (U = 359, *p*-value = 0.93) and third weeks (U = 426, *p*-value = 0.71). This analysis showed no significant differences. Therefore, for none of the groups did the contraction test offer clinically relevant information in a three-week period. 

## 4. Discussion

This study supplies strong evidence that an increase in daily TERT dose provides better results for improving PROM for PIPJ flexion contractures. The characteristics of the ETDNO make it a helpful device to increase daily TERT. Our results demonstrate that complete extension is a potential result in the treatment of PIPJ flexion contracture.

### 4.1. Daily TERT

All of the previous PIPJ flexion contracture studies with ETOs had used a daily TERT under twelve hours [4,5,17]. Following this study, we can refute the statement “it may not be clinically practical to expect patients to comply with a daily TERT beyond twelve to fourteen hours” [17]. 

Some patients had slight difficulties with ETDNO wear that impacted daily TERT adherence. These difficulties consisted of discomfort with ETDNO during the initial period of device use or when the patient transitioned to a new ETDNO. Group B patients had more incidence of discomfort compared to Group A. It appears that repeated doffing of the ETDNO for a few hours allowed the finger contracture to increase after hours without orthosis use. This then increased the ETDNO force when the patient ultimately donned it [16], creating some discomfort.

### 4.2. PROM Results

In this study, Group A achieved a mean improvement of 18° PROM in extension after the first week of intervention. We obtained a similar result (19°) in Group B, but at three weeks. Prosser (18°) and Glasgow (18.1° and 18.4°) obtained a similar result after eight weeks. Group A cumulatively improved by 29° at week three (Table 6). Results in Group A (M = 18.6, SD = 6.77, IC95%: 15.9–21.2) are statistically superior (U = 209, *p*-value = 0.002, IC95%: 2–10) to those obtained in Group B (M = 12.6, SD = 5.23, IC95%: 10.6–14.5)

**Figure 3:**
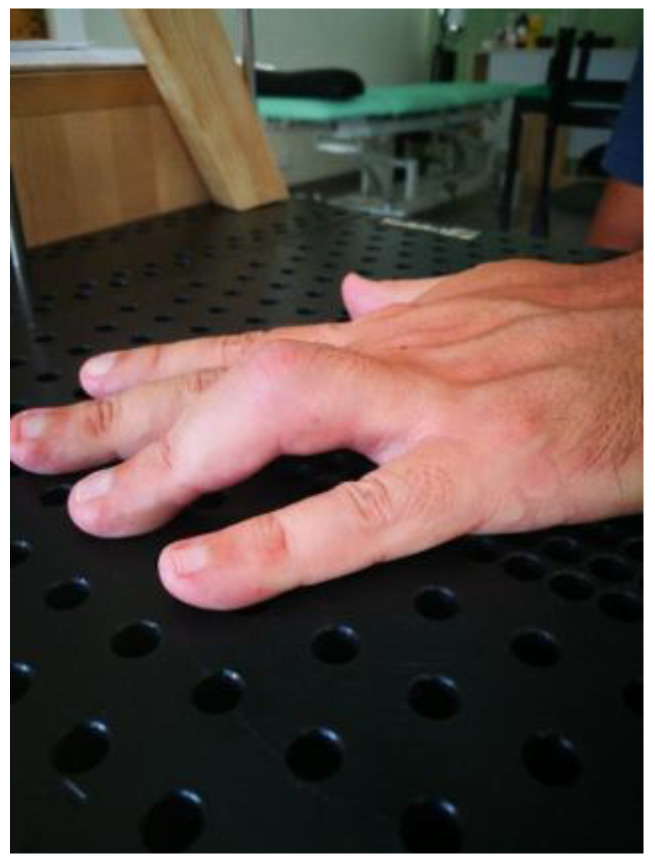
Flexion contracture after a strain at the proximal interphalangeal joint of the fourth finger complicated with Complex Regional Pain Syndrome of four-month progression treated in Group A.

**Figure 4:**
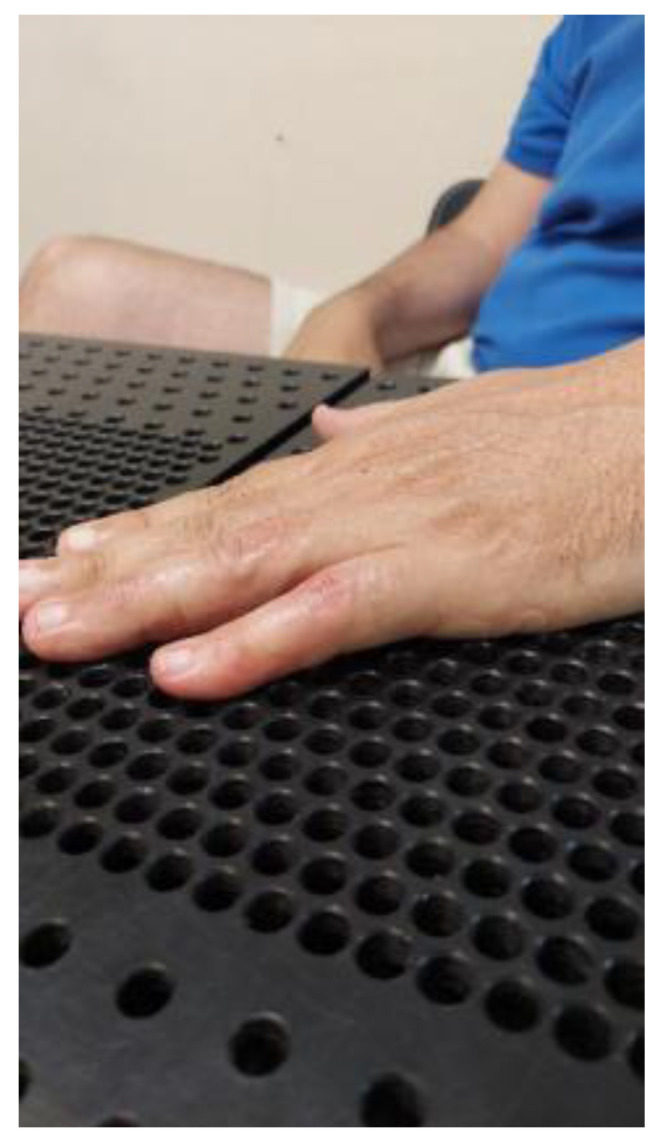
Complete extension of the proximal interphalangeal joint of the ring finger after three weeks of treatment.

**Figure 5:**
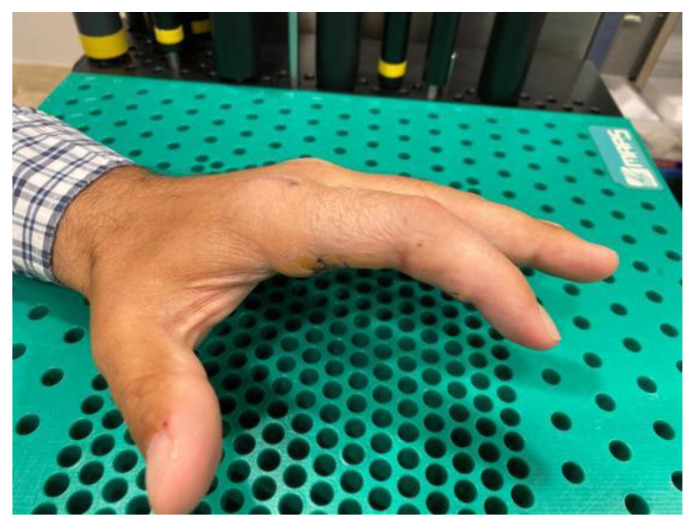
Flexion contracture at third months after teno-arthrolysis in a finger that is one year post flexor tendon repair. Pulley A2 was lost, and flexion contracture appeared again after the tenolysis. Treated in Group A.

**Figure 6:**
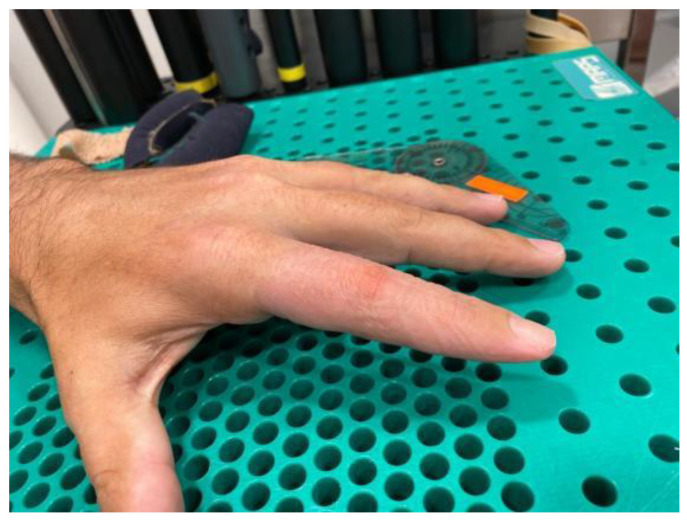
Complete extension of the proximal interphalangeal joint of the index finger after three weeks of treatment.

**Figure 7:**
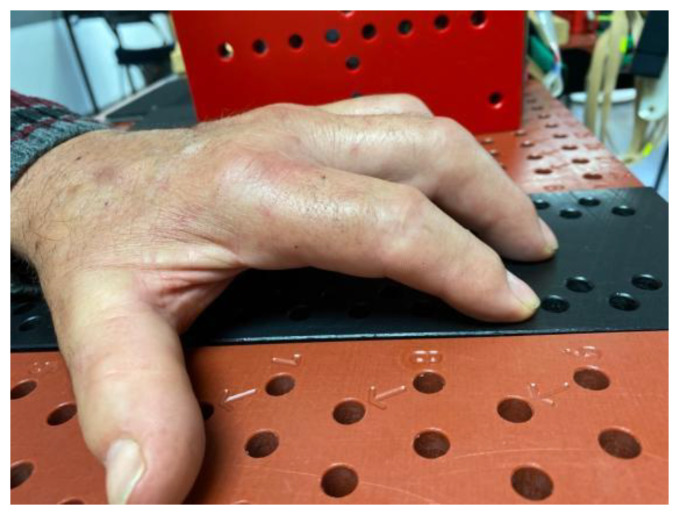
Six-month evolution of index and middle finger proximal interphalangeal joint flexion contracture following onset of Complex Regional Pain Syndrome after an amputation of the fifth finger. Treated in Group A.

**Figure 8:**
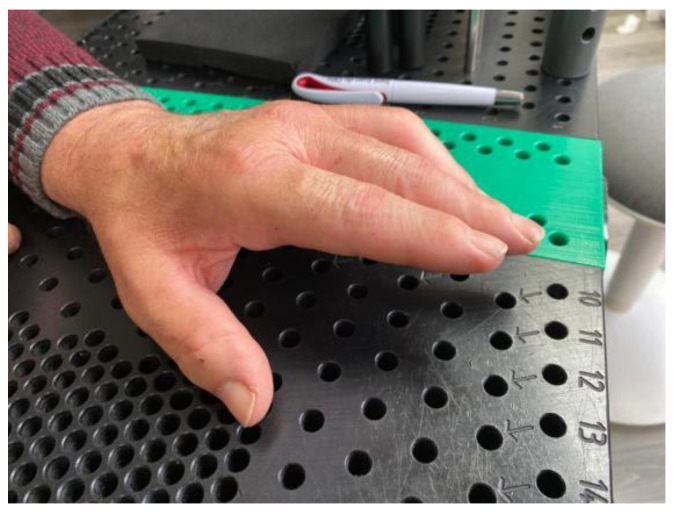
Complete extension of the proximal interphalangeal joint of the index and long fingers after three weeks of treatment.

**Figure 9:**
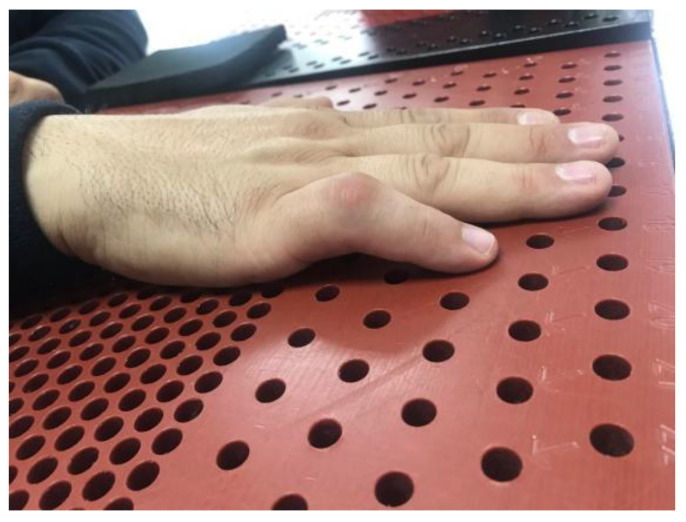
PIPJ flexion contracture fourth month after dislocation of the proximal interphalangeal joint. Treated in Group B.

**Figure 10:**
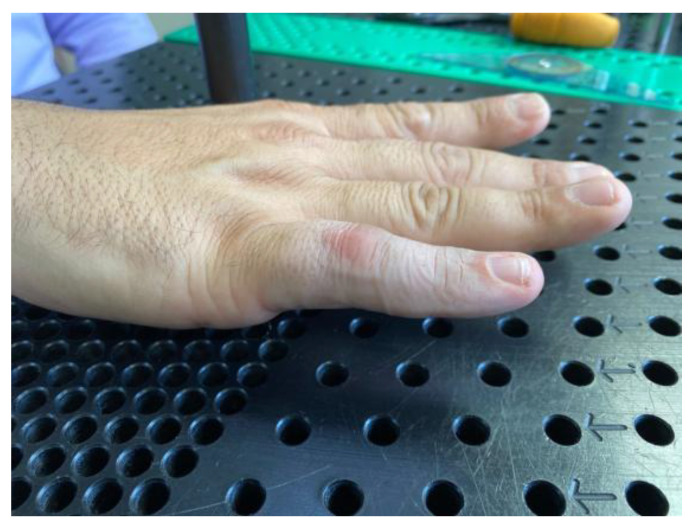
Complete extension of the proximal interphalangeal joint of the little finger after three weeks of treatment.

The literature has not previously described complete resolution (full extension) of PIPJ flexion contractures. Indeed, Prosser stated “No subject’s flexion contracture completely resolved” [4]. No subject in her study achieved a neutral PIP joint. In our study, seventeen subjects obtained 0° at the PIP joint, thirteen PIPJs in Group A (48%), and four PIPJs in group B (13.3%) (Figure 2, Figure 3, Figure 4, Figure 5, Figure 6, Figure 7, Figure 8, Figure 9 and Figure 10).

**Figure 11:**
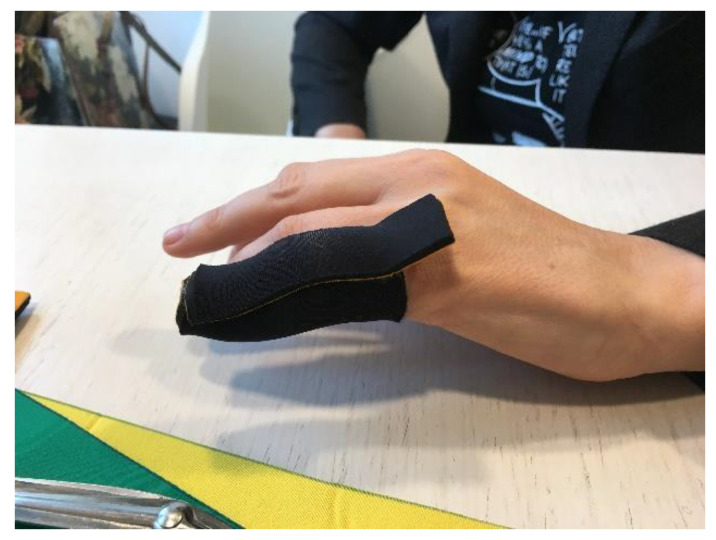
Elastic tension digital neoprene orthosis.

We know that to obtain a stable result, treatment can be as long as four-point-three months [4]. For this reason, as our follow-up period consisted of only three weeks, we will need another study with a longer follow up to determine the stability of the outcomes achieved using the ETDNO (Figure 11). 

Short, recurrent intervals of flexion exercise were enough, not only to avoid flexion PROM loses, but to improve them a little bit. We determined that the flexion loss that occurred in some patients was due to failure to remove the ETDNO and perform flexion exercises. 

### 4.3. Qualitative Measurements

In this study, the MWT was positive in all patients [6]. We found no relationship between the results of the MWT and the final outcome at the end of the third week. Some of the PIP joints demonstrated a greater change in PROM in response to the tension applied during the MWT. However, this behavior did not have value for the prognosis of the treatment. 

The contraction test showed that all patients’ PIPJs demonstrated a rapid PROM reaction following the release of tension upon removal of the ETDNO. In all cases, the PIPJs lost extension compared to the PROM measurement at the time of ETDNO removal. In neither group A, nor in group B, did we find differences in the contraction test results between the first and the third week. Additional research into this test will deepen our understanding of the treatment of PIPJ flexion contractures. 

### 4.4. ETDNO

Serial elastic tension treatment using an ETDNO method has demonstrated its effectiveness to take us further in the treatment of the finger flexion contractures with more rapid results and better PROM improvement. Patients with PIPJ flexion contractures will very likely benefit from ETDNO use. In our opinion, the unique design properties of the ETDNO made it possible to achieve higher daily TERT levels, exceeding twelve hours, than any study has ever before documented. We theorize that the previously applied devices generated too much force over too small a skin surface area. To definitively determine conclusions about forces and surface of force application will require further research. Studies that compare the ETDNO with other devices will help us understand the differences between orthosis approaches. 

## 5. Conclusions

This study provides powerful evidence for an approach to treat PIP flexion contractures. While we do not yet know the duration of treatment required to achieve a stable result with a PIPJ flexion contracture, we consider the results of this study as a recommendation for the type of device to use for PIP flexion contracture resolution and a daily TERT. As previously stated, the main weakness of this study is the short interval for follow up. Additional research will benefit from a longer follow up.

The results of this study heavily suggest that a dose of twenty hours of daily TERT with the joint in extension does not pose a risk for finger flexion loss if the patient exercises at least four times a day for twenty minutes. The patient who requires sustained extension treatment should always incorporate intermittent flexion exercises. 

This study shows evidence that, when treating PIPJ flexion contractures, an increase in daily TERT results in a commensurate PROM improvement. This research also demonstrates that an orthosis design, such as the ETDNO, increases the daily TERT when treating stiffness. In this study, serial elastic tension treatment using an ETDNO method achieved greater improvements in the amount of extension PROM increase compared to previous studies. The intervention also succeeded in shortening the time needed to obtain the result. In addition, we now know that conservative measures can accomplish complete extension. 

## Figures and Tables

**Table 1:** Pathologies in both groups.

	Group A	Group B
Sprain	9	11
Dislocation	6	6
Fracture	1	8
Flexor tendon	2	0
Surgical Dupuytren recurrence	2	0
Tumor	1	1
Complex Regional Pain Syndrome end stage	5	3
Infection	1	0
Ulnar nerve laceration	0	1
Total	27	30

**Table 2:** Daily TERT and difficulties in elastic tension digital neoprene orthosis use. Patients described discomfort the first day related to the tension that they perceived. After some hours of use, this tension decreased. It appeared that, because group B used the orthosis intermittently, they had recurrent difficulty with re-initiating the traction on the finger. This resulted in “more frequent reports of discomfort”.

Mean Daily Total End Range Time	20.5 h	12 h
Achieved the recommended range	81%	100%
Did not achieved the recommended range	14.8% patients 18 h 3.7% patient 19	
Maceration	3.7%	0%
Redness at proximal interphalangeal joint	3.7%	0%
Loss flexion	0%	0%
Discomfort the first day	18.51%	56.6%
Discomfort at night	18.51%	56.6%
Work-related problems	0%	6.66%

**Table 3:** Mean passive range of motion improvement in both groups.

	Group A	Group B
Total extension deficit in degrees	948°	914°
Mean previous time of contracture	9 m (SD: 2.07)	4 m (SD: 10.39)
Mean extension initial	35 (SD: 10.49)	29.2 (SD: 9.93)
Improvement° week 1	18	12
% Improvement week 1	501/948 (52.84%)	377/914 (41.24%)
Mean extension week 1	18.5	17.9
Improvement° week 3	29	19
% Improvement week 3	789/948 (83.22%)	570/914 (62.36%)
Mean extension final	5.8	11.5
Mann-Whitney U statistical test First week and third week	U = 238, *p*-value = 0.02	U = 156, *p*-value = 0.001
Mann-Whitney U statistical test Differences between groups	U = 229, *p*-value= 0.004	
Mean extension week 3	5.8	11.5
Daily TERT obtained	20.5 h	12 h
Mean flexion improvement	5.5°	4.2°

**Table 4:** Result classification at the end of the treatment.

Final Result	Flexion Contracture	Group A	Group B
Excellent	0°	13 (48.1%)	4(13.3%)
Good	0°–5°	4 (14.8%)	6 (20%)
Acceptable	5°–10°	3 (11.1%)	1 (3.3%)
Fair	10°–15°	3 (11.1%)	8 (26.6%)
Poor	>15°	4 (14.8%)	11 (36.6%)

**Table 5:** Modified Weeks test (MWT) and contraction test (CT) did not show statistical differences. SD= Standard deviation.

	First Day			Week One		Week Three	
MWT	Group A	Group B		Group A W1	Group A W3	Group B W1	Group B W3
MWT positive	100%	100%	Contraction test	5.7° (SD 2.30°)	5.4° (SD 3.69°)	6.2° (SD 2.57°	6.3° (SD 3.8°)
Mean MWT test	8° (0–12°)	7.5°					
MWT 0°–5°	6 fingers	4 fingers					
MWT 5°–10°	18 fingers	21 fingers	Mann-Whitney U	(U = 359, *p*-value = 0.93)		(U = 426, *p*-value = 0.71)	
MWT > 10°	3 fingers	5 fingers					
Mann-Whitney U	U = 350, *p*-value = 0.37						

**Table 6:** Results compared to other authors.

	Week 1	Week 3	Week 8
Punsola Group A Daily TERT 20.5 h	18°	29°	-
Punsola Group B Daily TERT 12 h	12°	19°	-
Prosser Group A 9.5 h	-	-	18°
Prosser Group B 11.5 h	-	-	
Glasgow group A 8 h	-	-	18.1°
Glasgow group B 11 h	-	-	18.4°

## Data Availability

Not applicable.
